# Inverse Associations between a Locally Validated Mediterranean Diet Index, Overweight/Obesity, and Metabolic Syndrome in Chilean Adults

**DOI:** 10.3390/nu9080862

**Published:** 2017-08-11

**Authors:** Guadalupe Echeverría, Emma E. McGee, Inés Urquiaga, Paulina Jiménez, Sonia D’Acuña, Luis Villarroel, Nicolás Velasco, Federico Leighton, Attilio Rigotti

**Affiliations:** 1 Center of Molecular Nutrition and Chronic Diseases, School of Medicine, Pontificia Universidad Católica de Chile, Santiago 8331150, Chile; gecheverria@bio.puc.cl (G.E.); emma.mcgee@nyu.edu (E.E.M.); iurquiaga@bio.puc.cl (I.U.); guadalupe.echeverria@gmail.com (P.J.); soniadacu@gmail.com (S.D.); fleighton@bio.puc.cl (F.L.); 2 Department of Public Health, School of Medicine, Pontificia Universidad Católica de Chile, Santiago 8331150, Chile; lv@med.puc.cl; 3 Department of Nutrition, Diabetes and Metabolism, School of Medicine, Pontificia Universidad Católica de Chile, Santiago 8331150, Chile; nvelasco@med.puc.cl

**Keywords:** Mediterranean diet, diet quality index, metabolic syndrome, obesity, overweight, chronic disease, Chile

## Abstract

Obesity and metabolic syndrome (MetS) are key risk factors for chronic disease. Dietary patterns are critical in the incidence and persistence of obesity and MetS, yet there is few data linking diet to obesity and MetS in Chile. Our objective was to use a locally validated diet index to evaluate adherence to a Mediterranean dietary pattern and its correlations with overweight/obesity (OW/O) and MetS prevalence in Chilean adults. We conducted a nationwide, cross-sectional online survey of Chilean adults with complete self-reported diet and body mass index data (*n* = 24,882). A subsample of 4348 users (17.5%) had valid MetS data. An inverse association was observed between adherence to Mediterranean diet and OW/O and MetS prevalence. As diet quality decreased from healthy, to moderately-healthy, to unhealthy, prevalence increased from 44.8, 51.1, to 60.9% for OW/O and from 13.4, 18.5, to 28.9% for MetS (*p*-values < 0.001). Adjusted odds ratios for OW/O and MetS were significantly higher in moderately-healthy (OR = 1.58 and 1.54) and unhealthy (OR = 2.20 and 2.49, respectively) diet groups in comparison to the healthy diet group. This study represents the first report on the relationship between Mediterranean diet and chronic disease risk in Chile. It suggests that the Mediterranean diet may be applied to manage chronic disease risk beyond the Mediterranean basin.

## 1. Introduction

Non-communicable chronic diseases are the leading cause of death globally, accounting for 38 million or 68% of total world deaths in 2012. Almost three quarters of these chronic disease-related deaths occur in low- and middle-income countries, making research on chronic disease risk factors in these countries of special importance [1].

Both obesity and metabolic syndrome (MetS)—a clustering of risk factors that increases chronic disease risk [2]—antedate and are associated with chronic disease. MetS correlates with and predicts several leading chronic conditions, including cardiovascular disease [3], type 2 diabetes [4], chronic kidney disease [5], and various cancers [6]. Direct associations between obesity and chronic diseases such as hypertension, dyslipidaemia, and ischemic heart disease have also been confirmed [7].

Among environmental factors affecting both obesity and MetS risk, dietary patterns have been shown to be of central importance [8,9]. Global evaluations of dietary patterns, rather than examinations of individual or small groups of nutrients and foods, are better able to capture real-world dietary practices as well as specific food combinations [10,11] and have been successfully used to assess the diet-disease relationship [12]. In fact, diet quality indices have been shown to be useful tools for measuring dietary patterns, identifying diet-related health outcomes, and creating guidelines and nutritional policies [13]. 

In particular, adherence to a Mediterranean dietary pattern emphasizing the use of olive oil as the principal source of fat; high intake of fruits, vegetables, legumes, whole grains, and nuts; moderate intake of low-fat and fermented dairy products, lean meat, fish, seafood, and red wine; and low intake of fatty and processed meats, sugar, and other processed foods [14], has repeatedly proven to be associated with lower incidence of both obesity [15,16,17] and MetS [18,19,20,21] in observational studies. Randomized intervention trials [22] and clinical studies [18,23,24] have also shown that a Mediterranean dietary pattern reverses and/or reduces MetS incidence. This has been shown for obesity incidence as well [25]. 

In Chile, chronic diseases are the principal cause of mortality, accounting for more than 60% of total deaths from 2000 to 2011 [26]. According to the nationally representative 2009–2010 Chilean National Health Survey, obesity (27.4%), overweight (39.3%), and MetS (35.3%) prevalence is alarmingly high [27]. Nevertheless, there is a dearth of high-quality studies investigating the link between dietary patterns and chronic disease risk in Chile [28,29,30,31,32]. Although findings from the 2012 Chilean National Survey for Food Consumption (ENCA) were recently published, associations between dietary patterns and clinical outcomes for chronic disease risk were not reported. A recent report based on the 2010 Chilean National Health Survey did, however, indicate that an abbreviated healthy diet index was inversely associated with MetS prevalence in Chilean adults [31]. 

Measurement of diet quality is also of concern in existing studies in Chile. The ENCA survey measured diet quality using a version of the US Healthy Eating Index previously adapted for use in Spain, rather than a diet index specifically tested and validated in Chile [28], while the 2010 Chilean National Health Survey employed a short, five-item food frequency questionnaire measuring only dairy, whole grain, fruit, vegetable, fish, and seafood consumption [27]. Scarcity of high-quality evidence, coupled with the growing epidemics of overweight/obesity (OW/O) and MetS, make the application of a locally validated diet quality index crucial to rapid, reliable, and reproducible identification of dietary correlations with chronic disease related outcomes in Chile. 

We propose the Mediterranean diet as a particularly fitting model for the analysis of Chilean diet quality because Central Chile, also known as the country’s agricultural heartland, represents one of the world’s five Mediterranean ecosystems. In addition, many traditional Chilean dishes, such as *charquicán*, *porotos granados*, *caldillo de congrio*, and *pebre*, incorporate ingredients and cooking techniques commonly used in a Mediterranean diet [33,34,35]. Evaluation of adherence to a Mediterranean dietary pattern may therefore prove useful in establishing associations with obesity and MetS incidence in Chile, and perhaps even in the evaluation of chronic disease risk and development of corresponding prevention and treatment initiatives [36].

Although many Mediterranean diet quality indices have been developed and successfully implemented in study populations around the world [13,37], until very recently none of these indices had been adapted or validated for use in Chile [38]. Most existing indices employ full-length food frequency questionnaires, time-consuming 24-h recalls, diet surveys composed of more than 100 items, or other complex tools [39], making them expensive and often complicated to implement. Yet, findings from the PREDIMED trial showed that a simple 14-item index for Mediterranean diet assessment provides robust, valid results [39]. We have developed the Chilean Mediterranean Diet Index (Chilean-MDI)—the first Mediterranean diet quality index to be adapted and validated specifically for use in Chile—taking into account local Chilean eating patterns and ingredients while relying on transferable criteria established by the Mediterranean dietary pattern [38]. The Chilean-MDI offers a short, easy-to-implement alternative to lengthier indices, in addition to being validated for online and self-assessment use [38]. However, no study has yet investigated clinical outcomes correlated with the Chilean-MDI, and very little research exists on the relationship between the Mediterranean dietary pattern, OW/O, and MetS in Chile.

In light of the importance of evaluating diet quality associations with chronic disease risk, especially in Chile where chronic disease prevalence is increasing and evidence is scarce, we used the Chilean-MDI as an indicator of adherence to a Mediterranean dietary pattern in order to evaluate its correlation with two important clinical risk factors for chronic disease—OW/O and MetS—among a sample of Chilean adults. 

## 2. Materials and Methods 

### 2.1. Study Design, Data Collection, and Subjects

We conducted a nationwide, cross-sectional survey using a self-selecting sample of Chilean internet-users. Data was collected via Fitbook, an online tool that offers adults the opportunity to self-evaluate their lifestyle and basic health indicators and receive individually tailored health recommendations based on their results. Fitbook is housed under the Aliméntate Sano website (www.alimentatesano.cl), an open-access platform that, in addition to Fitbook, also provides general health resources and information aimed at preventing chronic diseases in the Chilean population. The Fitbook survey consists of various questionnaires covering diet, physical activity, smoking, and other health-related habits, body mass index (BMI), clinical measurements of MetS, psychological well-being, and demographic characteristics. All data collection is self-reported and voluntary.

Beginning in August 2010, Aliméntate Sano and Fitbook were advertised throughout Chile via a comprehensive, nation-wide mass media campaign primarily utilizing internet and radio promotion in combination with printed press and television coverage. Today, the Fitbook registry includes self-evaluated lifestyle and health data from more than 70,000 voluntary adult internet users. 

**Figure 1:**
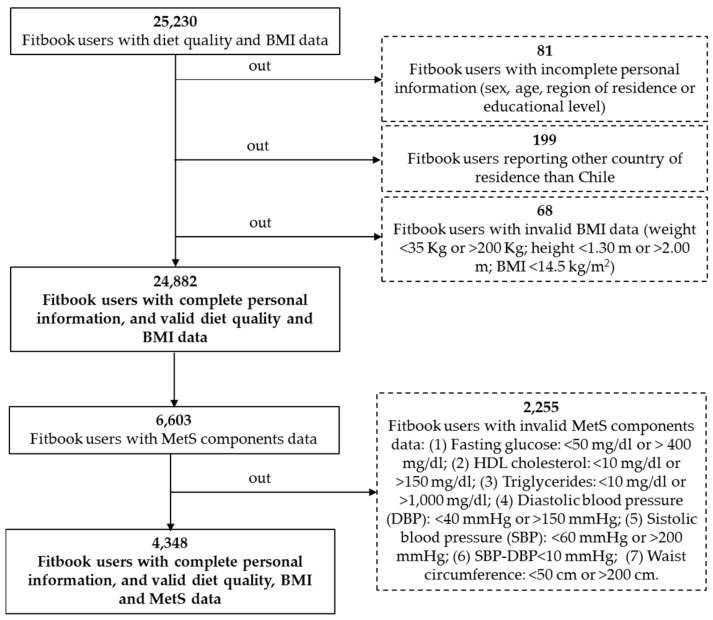
Flow chart of Chilean adults included in this study: 24,882 with valid data for diet quality and BMI, and a subsample of 4348 with complete and valid data for MetS.

For the present study, we used Fitbook user data collected from its inception in August 2010 through October 2015. The self-selecting sample consisted of all voluntary Chilean Fitbook users aged 19 or older with complete diet quality and BMI data after validation (*n* = 24,882). Of this total sample, a subsample of 4348 users (17.5%) had complete, valid MetS data, as shown in Figure 1.

Although Fitbook allows users to complete the survey more than once and thus track their progress over time, for the purpose of this study we have included only baseline survey data. All follow-up surveys (repeat surveys by the same user) have been excluded from our analyses.

All subjects gave their informed consent for inclusion before they participated in the study. The study was conducted in accordance with the Declaration of Helsinki, and the protocol was approved by the Ethics Committee of the School of Medicine at Pontificia Universidad Católica de Chile.

### 2.2. Dietary, Anthropometric, and Clinical Measurements

The primary variables of interest for this study were: (i) diet quality according to the Chilean-MDI; (ii) BMI status according to self-reported height and weight; and (iii) MetS presence according to self-reported measurements for each of the five MetS components.

The Chilean-MDI is a 14-item index composed of 22 multiple-choice questions that measure adherence to a Mediterranean diet [38]. The index was developed from a previous validated Mediterranean eating score, which was then adapted to reflect Chilean dietary habits [36] and further validated by comparing its normal distribution (Kolmogorov–Smirnov test) and concordance (Spearman correlation, Bland–Altman, and kappa coefficient tests) between results obtained by a trained nutritionist versus online self-application [38]. The Chilean-MDI evaluates 14 food groups, each of which is assigned a score of 0, 0.5, or 1 depending on reported average consumption frequencies and their beneficial or detrimental health effects as defined a priori. Overall index scores range from 0 to 14 points, representing minimum to maximum Mediterranean diet adherence, respectively. Scores from 0 to 4.5 were classified as unhealthy, while scores from 5 to 8.5 were considered moderately healthy and scores from 9 to 14 were considered healthy. Food groups and consumption level scores are shown in Table 1. The application of this index in Chilean adults showed a better diet quality—i.e., high Mediterranean diet adherence—among women, with advanced age, and among people with higher educational levels, demographic trends that are comparable to those obtained with other Mediterranean indexes applied in Europe and North American populations [13,37,39]. Documentation of Chilean-MDI development and validation is described in further detail elsewhere [36,38].

BMI was calculated using self-reported height and weight measurements. Individuals were categorized according to standard weight status cutoff points for adults: underweight (<18.5 kg/m^2^), normal weight (18.5–24.9 kg/m^2^), overweight (25–29.9 kg/m^2^), and obese (≥30 kg/m^2^). The same cutoff points were used for both men and women. 

MetS was evaluated according to self-reported measurements of its five individual components. Cutoff points used were those proposed by the American Heart Association update to the Adult Treatment Panel III (ATP-III) criteria [40]: (i) abdominal obesity (waist circumference > 102 cm in men or >88 cm in women); (ii) low HDL-cholesterol (<40 mg/dL in men or <50 mg/dL in women, or on drug treatment); (iii) high triglycerides (≥ 150 mg/dL, or on drug treatment), (iv) blood hypertension (blood pressure ≥ 130/85 mmHg, or on antihypertensive drug treatment); and (v) high blood glucose (fasting plasma glucose concentration ≥ 100 mg/dL, or on drug treatment for elevated glucose). According to these criteria, presence of three or more of any of the five MetS components resulted in a positive diagnosis.

BMI and MetS data were cleaned, excluding clinically invalid or corrupt data points. Cutoff points for invalid data were determined using results from the 2010 nationally-representative Chilean National Health Survey [27].

Additional variables in our analysis included sex, age, smoking status, and physical activity. Physical activity was evaluated according to the Spanish version of the short-form, self-administered International Physical Activity Questionnaire (IPAQ) for young and middle aged adults. We also analyzed data on education level (years of school completed) and region of residence.

### 2.3. Statistical Analyses

Univariate, bivariate, and multivariate analyses were conducted. Student *t*, Chi-squared, one-way ANOVA, and Tukey’s HSD tests were performed. Multivariate, binomial logistic regression models were created for both OW/O and MetS presence. Odds ratios and their corresponding confidence intervals were calculated from the regression models. Odds ratios for each individual MetS component and disaggregated food groups were also calculated from binomial logistic regression models. All logistic regression models were adjusted for sex (dichotomous), age (continuous), years of school completed (dichotomous: ≤12 or >12 years of formal education), smoking status (categorical: non-smoker, moderate smoker (≤5 cigarettes/day), or heavy smoker (>5 cigarettes/day), physical activity (categorical: high, medium, or low based on IPAQ), and, in the case of MetS only, BMI (continuous). Analyses were conducted in *R* [41], IBM SPSS Statistics for Windows, Version 22.0. Armonk, NY, USA: IBM Corp., and Microsoft Excel 2011.

## 3. Results

### 3.1. Sample Characteristics

Our sample of Chilean adults (*n* = 24,882) was primarily composed of women (68.5%), young adults (43.1% aged 19 to 29 years old, mean age = 34.21, SD = 11.36, range = 19 to 85), highly educated individuals (82.1% completed more than 12 years of formal education), and residents of Greater Santiago and its surroundings (57.5%) (Table 2). Average BMI was 26.10, SD = 4.61, and both obesity (17.9%) and overweight (35.9%) prevalence was high. In addition, nearly 38% of the sample reported current smoking and 39% of respondents reported low levels of physical activity.

In the sub-sample of individuals with complete, valid MetS data (*n* = 4348), 20.0% were MetS positive (three or more components surpassing ATP-III cutoff points) and 76.2% presented at least one MetS component. In the subsample that exhibited MetS, 36.1% of participants exhibited abdominal obesity, 24.5% reported low HDL-cholesterol, 29.2% had high triglycerides, 10.7% were hypertensive, and 26.2% displayed high blood glucose.

### 3.2. Diet Quality Evaluation Using Chilean-MDI

Chilean-MDI scores ranged from zero to 14 points, with a mean score of 5.71 (95% CI (5.68, 5.74)). Only 8.5% of the sample had a diet score that qualified as healthy or high Mediterranean diet adherence (9 to 14 points), while 57.0% had a moderately healthy score (5 to 8.5 points) and 34.5% had an unhealthy score (0 to 4.5 points).

Men had lower mean Chilean-MDI scores than women (5.60 vs. 5.76, Welch two sample *t*-test *p*-value < 0.001), while respondents who had completed more than 12 years of school had higher scores than respondents who had completed 12 years or less (5.91 vs. 5.13, Welch two sample *t*-test *p*-value < 0.001). By region, respondents who resided in Greater Santiago and its surroundings had higher scores compared to respondents living in other regions (5.87 vs. 5.49, Welch two sample *t*-test *p*-value < 0.001).

A significant increase in mean diet score was noted in older age groups: mean Chilean-MDI score was 5.23 for respondents aged 19–29, 5.72 for ages 30–39, 6.16 for ages 40–49, 6.76 for ages 50–59, and 7.49 for ages 60 or older (one-way ANOVA *p*-value < 0.001, Tukey’s HSD adjusted *p*-values < 0.001 for comparisons between all age groups). Mean diet index score also increased as physical activity level rose from low to moderate to high (5.12 vs. 5.97 vs. 6.58, ANOVA *p*-value < 0.001, Tukey’s HSD adjusted *p*-values < 0.001 for all comparisons). Finally, mean Chilean-MDI scores were lower for heavy smokers than they were for moderate and non-smokers (5.22 vs. 5.38 vs. 6.05, ANOVA *p*-value < 0.001, Tukey’s HSD adjusted *p*-values < 0.01 for all comparisons). 

### 3.3. Associations between Chilean-MDI, BMI, and MetS 

The mean Chilean-MDI score for OW/O respondents was significantly lower than the mean score for normal/underweight respondents (5.48 vs. 5.98, 95% CI for difference in means (0.44, 0.55), Welch two sample *t*-test *p*-value < 0.001). The mean diet index score for MetS positive respondents was also lower than the mean score for MetS negative respondents (5.88 vs. 6.64, 95% CI for difference in means (0.61, 0.92) Welch two sample *t*-test *p*-value < 0.001). 

**Figure 2:**
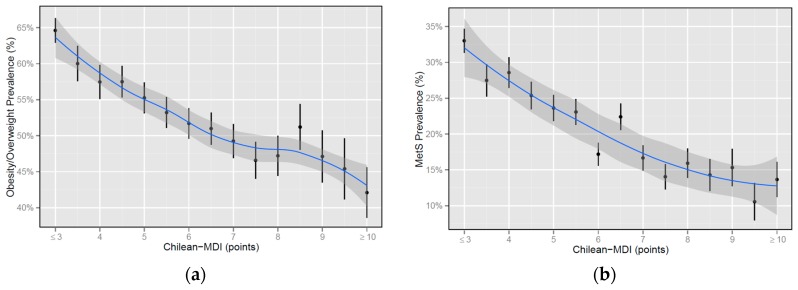
Overweight/obesity (**a**) and metabolic syndrome (**b**) prevalence by Chilean-MDI score. Standard error bars are shown for each data point. Prevalences were aggregated at end points due to small sample sizes. Smooth conditional prevalence was approximated by the curve with standard errors shaded in gray.

In our sample of Chilean adults, unadjusted OW/O and MetS prevalence exhibited a strong, monotonic inverse relationship with Chilean-MDI score (Figure 2a,b, respectively). Crude prevalence for both outcomes was very strongly correlated with Chilean-MDI score (Spearman’s rho = −0.95 and −0.96 for OW/O and MetS, respectively). Correlations between individual MetS component prevalence and Chilean-MDI score were very strong to moderate (Spearman’s rho = −0.89 for abdominal obesity, −0.84 for blood hypertension, −0.82 for high blood glucose, −0.73 for low HDL cholesterol, and −0.59 for high triglycerides), with the strongest correlations observed for abdominal obesity, blood hypertension, and high blood glucose, although no single MetS component had a stronger correlation coefficient than MetS or OW/O. All correlation coefficients were statistically significant (*p*-values < 0.05). In categorical terms, OW/O prevalence increased from 44.8% in the healthy diet group, to 51.0% in the moderately healthy group, to 60.6% in the unhealthy group (Pearson’s Chi-squared = 271.48, *p*-value < 0.001). MetS prevalence behaved similarly, increasing from 13.4% in the healthy diet group, to 18.5% in the moderately healthy group, to 28.9% in the unhealthy group (Pearson’s Chi-squared = 71.417, *p*-value < 0.001). 

**Figure 3:**
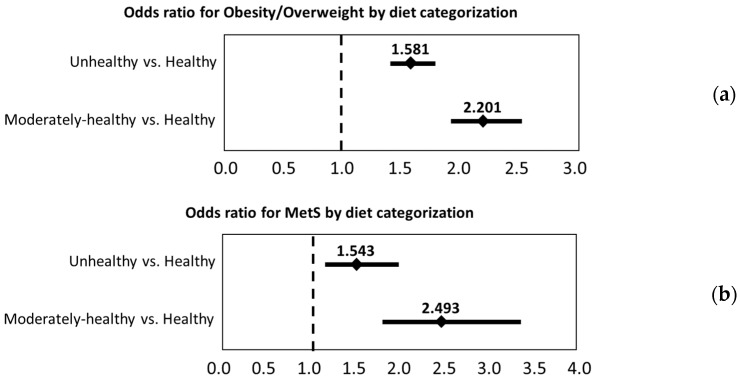
Multivariate adjusted odds ratios and 95% CI for obesity/overweight (**a**) and metabolic syndrome (**b**) by Chilean-MDI diet category. Odds ratios were adjusted by sex, age, years of school completed, smoking status, and physical activity.

Binary logistic regression models were used to test for the robustness of the diet–disease relationships after adjusting for control variables. Multivariate adjusted odds ratios (shown in Figure 3a,b) for both OW/O and MetS were calculated from binary logistic regression. Moderately healthy and unhealthy (i.e., moderate and low adherence to a Mediterranean diet) groups were compared to the healthy (i.e., high adherence to a Mediterranean diet) group. In this sample of Chilean adults, odds for OW/O were significantly higher for individuals in both moderately healthy (OR = 1.58, 95% CI (1.41, 1.77)) and unhealthy (OR = 2.20, 95% CI (1.94, 2.50)) groups in comparison to those in the healthy group, even when adjusting for control variables. The adjusted odds for MetS were also significantly higher for moderately healthy (OR = 1.54, 95% CI (1.19, 2.01)) and unhealthy (OR = 2.49, 95% CI (1.83, 3.39)) groups in comparison to those in the healthy group.

### 3.4. Association between Chilean-MDI Food Groups, BMI, and MetS Components

Disaggregated analyses of the association between each of the 14 Chilean-MDI food groups with individual MetS components and OW/O revealed component-level diet-disease trends. Binary logistic regression models were created using healthy vs. unhealthy consumptions of individual food groups to predict the presence or absence of each MetS component and OW/O. Two models were used: Model 1 incorporated food groups individually in separate models, while Model 2 incorporated all food groups in the same model, controlling for consumption across all remaining food groups. Both models were adjusted for sex, age, years of school completed, physical activity, and smoking status. Table 3 shows the adjusted odds ratios for abdominal obesity, blood hypertension, elevated blood glucose, high triglycerides, low HDL-cholesterol, and OW/O.

A total of 42 significant relationships were identified, of which 18 relationships (42.9%) were confirmed by both Models 1 and 2; the remaining relationships were significant in only one of the two models. Non-concurrence between models is likely due to the large number of food groups used as controls in Model 2 and an accumulation of weak to moderate associations between food groups. The Goodman–Kruskal Gamma statistic measuring association between ordinal variables was 0.538 (95% CI (0.523, 0.554)) for vegetables and fruits, 0.317 (95% CI (0.3, 0.333)) for whole grains and low fat/fermented dairy products, and 0.419 (95% CI (0.404, 0.434)) for fatty/processed meats and sugar, for example.

Almost all associations between Chilean-MDI food groups, MetS components, and OW/O were consistent with findings from previous studies [39]. Low consumption of vegetables, legumes and nuts, fruits, whole grains, fish and seafood, low-fat and fermented dairy products, olive oil, and canola oil were associated with increased odds for one or more outcome. Over consumption of fatty and processed meats, whole fat dairy products, and sugar, along with occasional or excessive wine consumption, were also associated with increased odds for at least one outcome. 

Across both Models 1 and 2, disaggregated analyses of Chilean-MDI showed that over consumption of sugar, low consumption of nuts, and low consumption of low fat and fermented dairy products was associated with increased odds for abdominal obesity. Over consumption of sugar along with under consumption of vegetables, fish and seafood, and olive oil/healthy fats was associated with increased odds for hypertension. Low consumption of low-fat and fermented dairy products was associated with increased odds for elevated blood glucose, as was high consumption of fatty and processed meats, whole fat dairy products, and sugar. Over consumption of sugar and fatty/processed meats, occasional or excessive consumption of wine, and low consumption of legumes, nuts, whole grain cereals, and olive oil/healthy fats were associated with increased odds for obesity/overweight. In contrast, odds ratios for high triglycerides and low HDL-cholesterol were not significant across both models for any food group. 

Only one food group, lean meat and poultry, did not associate as predicted. Unhealthy consumption of lean meat and poultry was associated with decreased odds of abdominal obesity in Model 2, whereas for other MetS components and OW/O, unhealthy consumption was associated with increased odds for each outcome. Healthy consumption of lean meat and poultry was defined as 4–8 portions per week, while <4 or >8 portions per week was considered unhealthy. The reverse association observed for abdominal obesity is therefore likely due to the mixing that occurs in the unhealthy consumption group, where individuals who consumed no lean meat are combined with those who consumed an excessive amount. 

## 4. Discussion

This study reports the relationship between diet quality and chronic disease risk in Chile, where these conditions are increasing at alarming rates and existing research is limited. Our study provides a vital first step towards improved understanding of diet–disease relationship in Chile and is also the first Chilean study to use a locally validated Mediterranean diet index in evaluating cross sectional associations with OW/O and MetS. 

In this cross sectional sample, Chilean adults demonstrate high prevalence of obesity (17.9%), overweight (35.9%), and MetS (20.0%)—a finding that is consistent with evidence from a previous national survey [27]—as well as a relatively low Chilean-MDI score (mean = 5.71), with only 8.5% of the sample qualifying within the healthy diet/high Mediterranean diet adherence category. Mean Chilean-MDI scores were significantly lower for adults presenting OW/O and MetS than those not presenting these conditions. In fact, the Chilean-MDI was able to detect a strong, monotonic inverse association between adherence to a high quality, Mediterranean dietary pattern and both OW/O and MetS prevalence. We also found component level associations between individual food groups, MetS components, and OW/O. These associations cannot be explained by differences in sex, age, years of school completed, smoking status, or physical activity. Taken together, these findings suggest that low adherence to a Mediterranean style diet may be an important factor in increased chronic disease risk in Chile.

Though this study provides an important contribution to research on the diet-chronic disease relationship in Chile, several potential limitations exist. Our self-selecting sample of adult internet users is not representative of the Chilean population and, as a result of the study’s methodology, our results are not generalizable to the entire country. Our sample illustrates a self-selecting bias based on gender, age, socio-economic status, and region of residence. Specifically, women, young adults, educated individuals, and residents of Greater Santiago and its surroundings are all over represented in our sample. We also recognize an implicit bias towards internet-literate adults. 

All variables—including clinical MetS and BMI measurements—are self-reported, which may impact validity. Under-reporting and misreporting of dietary components in self-reported data has been documented and in some cases may result in spurious diet–disease relationships [42]. The scope of this study did not allow for confirmation of self-reported data points via clinical testing, detailed dietary monitoring, or biological markers. Nevertheless, our analysis relies primarily on the interpretation of global dietary patterns rather than the influence of individual foods, which have been shown to be more susceptible to underreporting [42]. The inverse associations between Mediterranean diet adherence and OW/O and MetS observed in this study have been confirmed in studies of varying methodological design, scope, and population, suggesting that the relationships we report here are likely not spurious. However, future studies should consider additional data validation methods in order to more accurately measure food consumption and directly evaluate nutritional status parameters.

Although we have a large total sample size of nearly 25,000 respondents, small samples within some subgroups may limit our ability to detect significant relationships. In the case of the MetS subsample, sample sizes for rarely consumed food groups and for extreme diet scores are quite small and may not be large enough to detect significant associations. The influence of relatively rare food groups (such as olive oil, which is not frequently consumed in Chile) is difficult to assess in our sample. Results from the disaggregated analyses of food groups should therefore be interpreted with caution.

Finally, although many dietary indices—including a 14-item tool used in the Spanish PREDIMED trial [39]—have been successfully validated for the assessment of associations between a Mediterranean dietary pattern and various health outcomes, researchers have identified the need for a more precise, quantitative definition of the Mediterranean diet in order to ensure accurate measurements across indices and allow for intra-study comparisons [13,43]. It is also important, however, to apply indices that are suited for specific local environments, given the heterogeneity of dietary patterns across the world. The locally validated Chilean-MDI offers a simple tool suited to the Chilean ecosystem and diet, while relying on the key components found in many Mediterranean diet indices [38]. Additional studies are needed to demonstrate associations with and the predictive value of Chilean-MDI for diabetes, cardiovascular disease, and cognitive decline, as well as to compare Chilean-MDI performance with the performance of other Mediterranean diet indices. Indeed, we hope to corroborate these findings in ongoing epidemiological studies (ELANS [44] and MAUCO [45]) in Chile, in which samples are more representative of the Chilean population and anthropometric and food intake, as well as biochemical markers, will be directly evaluated by health professionals or measured in validated clinical laboratories. 

In spite of these methodological limitations, our results are consistent with findings from other studies, including cross-sectional and longitudinal [18,39,46,47] as well as clinical intervention [22,48,49] studies, which have demonstrated a consistent relationship between adherence to a Mediterranean style diet and decreased prevalence of OW/O, abdominal obesity, and MetS. Our results not only corroborate findings from other regions, but also demonstrate that a Mediterranean diet is possible in Chile and, as in other countries, is associated with decreased risk of chronic disease conditions.

The high level of consistency in the diet index–chronic disease risk relationship reported in this study indicates that increased adherence to a Mediterranean dietary pattern among Chilean adults may help decrease chronic disease risk. In addition, for both OW/O and MetS, the difference in mean dietary index score between groups with and without each condition was less than 1 point on the 14 point Chilean-MDI scale, suggesting that even small improvements in diet quality may result in a decrease in OW/O and MetS incidence. This conclusion seems likely, given that Mediterranean diet has shown beneficial metabolic and clinical event effects in the PREDIMED randomized trial after 1–2 points increased adherence to a Mediterranean dietary pattern [50,51,52]. Further research should be conducted in order to determine if active promotion of adherence to a Mediterranean style diet could help decrease chronic disease risk in Chile as well as other Latin American countries. 

## Figures and Tables

**Table 1:** Chilean Mediterranean Diet Index (Chilean-MDI) definition and scoring [38].

	Score Item	Score Units	Scoring		
			1.0 Point	0.5 Points	0 Points
1	Vegetables (excluding potatoes)	Servings per day	≥3	1 to <3	<1
2	Legumes	Servings per week	>2	1 to 2	<1
3	Nuts	Servings per week	>2	1 to 2	<1
4	Fruits	Servings per day	≥2	1 to <2	<1
5	Whole grain cereals	Servings per day	≥2	1 to <2	<1
6	Lean meat and poultry	Servings per week	5–8	1–4	<1 or >8
7	Fish and seafood	Servings per week	>2	1 to 2	<1
8	Fatty and processed meats	Servings per week	<1	1 to 2	>2
9	Whole fat dairy products, not fermented	Servings per day	<1	1 to <2	≥2
10	Low fat and fermented dairy products	Servings per day	≥2	1 to <2	<1
11	Olive oil	Teaspoons per day	≥3	1 to <3	<1
12	Other healthy fats: Canola oil (C) and avocado (A)	C: consumption pattern A: units per week	regularly >3	occasionally 0.5 to 3	Never <0.5
13	Sugar (S) and sugary snacks, juices, and soft drinks (SS)	S: Teaspoons per day SS: Serving per day	<4 non-daily	<4 daily	≥4 daily
14	Wine	Glasses per day	1 to 2 regularly, usually with meals	<1 or >2 regularly, usually with meals	Non-drinker or irregular and usually not with meals

**Table 2:** Summary statistics of the self-selecting sample of Chilean adults shown by nutritional status and metabolic syndrome *.

Parameters	By Nutritional Status (*n* = 24,882)				By Metabolic Syndrome Presence (*n* = 4348)			
	Total	Under & Normal Weight	Overweight & Obesity	*p*-Value	Total	Without MS	With MS	*p*-Value
**Sex**								
Female	68.5%	78.5%	59.9%	<0.001	70.6%	72.2%	64.4%	<0.001
Male	31.5%	21.5%	40.1%		29.4%	27.8%	35.6%	
**Age**								
19–29 years old	43.1%	53.2%	34.4%	<0.001	33.5%	35.6%	25.3%	<0.001
30–39 years old	31.4%	28.6%	33.9%		29.0%	30.1%	24.4%	
40–49 years old	14.9%	11.0%	18.2%		17.8%	17.1%	20.8%	
50–59 years old	7.1%	5.0%	9.0%		11.5%	10.2%	16.7%	
>60 years old	3.5%	2.2%	4.6%		8.2%	7.0%	12.9%	
**Region of residence**								
Santiago metropolitan region	57.5%	59.2%	56.0%	<0.001	58.1%	58.8%	55.1%	<0.001
All other regions	42.5%	40.8%	44.0%		41.9%	41.2%	44.9%	
**Educational levels**								
≤12 years of school completed	17.9%	16.0%	19.5%	<0.001	14.6%	13.1%	20.6%	<0.001
>12 years of school completed	82.1%	84.0%	80.5%		85.4%	86.9%	79.4%	
**Smoking status**								
Non-smoker	62.1%	64.6%	60.1%	<0.001	70.3%	70.9%	67.7%	0.004
Moderate smoker	28.0%	27.3%	28.6%		21.4%	21.5%	21.0%	
Heavy Smoker	9.9%	8.1%	11.4%		8.3%	7.6%	11.2%	
**Physical activity level**										
Low	39.4%	32.8%	45.0%	<0.001	37.8%	34.6%	50.6%	<0.001
Medium	36.5%	38.4%	34.9%		38.1%	38.5%	36.8%	
High	24.1%	28.8%	20.1%		24.1%	26.9%	12.6%	
**BMI status**								
Underweight	1.4%	3.1%	0.0%	<0.001	0.9%	1.0%	0.2%	<0.001
Normal weight	44.7%	96.9%	0.0%		40.8%	48.2%	11.1%	
Overweight	35.9%	0.0%	66.7%		37.3%	36.9%	39.0%	
Obese	17.9%	0.0%	33.3%		21.0%	13.8%	49.7%	
**Number of MetS components present**								
0 components	23.8%	40.2%	12.1%	<0.001	23.8%	29.8%	0.0%	<0.001
1–2 components	56.2%	54.4%	57.5%		56.2%	70.2%	0.0%	
3–5 components	20.0%	5.5%	30.4%		20.0%	0.0%	100.0%	
**Chilean-MDI scoring**								
Unhealthy/low adherence to Mediterranean diet (0–4.5 points)	34.5%	29.4%	38.9%	<0.001	22.4%	19.9%	32.4%	<0.001
Moderately healthy/moderate adherence to Mediterranean diet (5–8.5 points)	57.0%	60.4%	54.1%		61.3%	62.4%	56.7%	
Healthy/high adherence to Mediterranean diet (9–14 points)	8.5%	10.1%	7.1%		16.3%	17.6%	10.9%	

* The total sample size was 24,882 for nutritional status, and 4348 for MetS; however, sample size varies slightly between parameters depending on the completeness of data collected for each variable.

**Table 3:** Adjusted odds ratios for metabolic syndrome components and obesity/overweight by individual Chilean-MDI food groups.

Food Groups	Intake Categories *	Statistical Model ^†^	Abdominal Obesity	Blood Hypertension	High Blood Glucose	High Triglycerides	Low HDL-Cholesterol	Obesity/Overweight
**Vegetables (excluding potatoes)**	<3 portions/day vs. ≥3 portions/day (ref)	Model 1	1.38 (1.16–1.65)	1.31 (1.09–1.56)	1.26 (1.04–1.53)			1.26 (1.15–1.37)
		Model 2		1.21 (1.00–1.46)				
**Legumes**	<2 portions/week vs. ≥2 portions/week (ref)	Model 1				1.51 (1.04–2.18)		1.51 (1.27–1.80)
		Model 2						1.24 (1.04–1.48)
**Nuts**	<2 portions/week vs. ≥2 portions/week (ref)	Model 1	1.79 (1.45–2.21)	1.34 (1.10–1.63)	1.36 (1.10–1.69)	1.28 (1.04–1.57)		1.504 (1.36–1.67)
		Model 2	1.58 (1.27–1.96)					1.35 (1.21–1.50)
**Fruits**	<2 portions/day vs. ≥2 portions/day (ref)	Model 1	1.30 (1.10–1.54)					1.22 (1.12–1.32)
		Model 2						
**Whole grain cereals**	<2 portions/day vs. ≥2 portions/day (ref)	Model 1	1.26 (1.07–1.49)	1.18 (1.00–1.39)				1.16 (1.08–1.25)
		Model 2						1.11 (1.03–1.20)
**Lean meat and poultry**	<4 or>8 portions/week vs. 4–8 portions/week (ref)	Model 1						
		Model 2	0.76 (0.61–0.93)					
**Fish and seafood**	<1 portion/week vs. ≥1 portion/week (ref)	Model 1	1.25 (1.08–1.45)	1.31 (1.13–1.52)	1.22 (1.04–1.43)			1.12 (1.05–1.20)
		Model 2		1.12 (1.03–1.40)				
**Fatty and processed meats**	≥1 portion/week vs. <1 portion/week (ref)	Model 1	1.30 (1.12–1.50)		1.41 (1.20–1.66)	1.19 (1.02–1.39)		1.47 (1.37–1.57)
		Model 2			1.21 (1.03–1.44)			1.37 (1.28–1.47)
**Whole fat dairy products, not fermented**	≥1 portion/day vs. <1 portion/day (ref)	Model 1	1.34 (1.15–1.57)	1.26 (1.08–1.47)	1.52 (1.29–1.80)			1.13 (1.06–1.21)
		Model 2			1.296 (1.086–1.546)			
**Low fat and fermented dairy products**	≤1 portion/day vs. >1 portion/day (ref)	Model 1	1.33 (1.15–1.55)	1.21 (1.05–1.41)	1.38 (1.18–1.62)			1.16 (1.09–1.24)
		Model 2	1.19 (1.02–1.39)		1.25 (1.06–1.48)			
**Olive oil and healthy fats (canola oil and avocado)**	≤3 tea-spoon/day vs. >3 tea-spoon/day (ref)	Model 1		1.43 (1.17–1.74)			1.34 (1.01–1.77)	1.29 (1.17–1.43)
		Model 2		1.30 (1.06–1.59)				1.12 (1.01–1.24)
**Wine**	Occasional or excessive vs. regular, moderate consumption (1–2 drinks/day) (ref)	Model 1						1.32 (1.17–1.49)
		Model 2					1.33 (1.01–1.76)	1.27 (1.13–1.43)
**Sugar**	Excessive consumption vs. moderate consumption (ref)	Model 1	1.47 (1.27–1.71)	1.36 (1.17–1.58)	1.47 (1.25–1.72)			1.32 (1.23–1.41)
		Model 2	1.28 (1.09–1.50)	1.24 (1.06–1.46)	1.23 (1.04–1.47)			1.15 (1.07–1.24)

* All OR were calculated using the healthy intake score as the reference category (ref). **^†^** Model 1: binary logistic regression model adjusted for sex, age, years of school completed, physical activity, and smoking status. Model 2: binary logistic regression model adjusted for sex, age, years of school completed, physical activity, smoking status, and food groups. Empty cells indicate non-statistically significant OR.
